# Embedding peer assessment in MCQs has improved immediate performance but increased task duration without enhancing transfer

**DOI:** 10.3389/fpsyg.2025.1659735

**Published:** 2025-10-01

**Authors:** Jean-François Parmentier, Kevin Sigayret, Franck Silvestre

**Affiliations:** ^1^IRIT, Université de Toulouse, CNRS, Toulouse INP, IPSA, Toulouse, France; ^2^IRIT, Université de Toulouse, CNRS, Toulouse INP, Université Toulouse Capitole, Toulouse, France; ^3^IRIT, Université de Toulouse, CNRS, Toulouse INP, Toulouse, France

**Keywords:** peer assessment, multiple-choice questions, asynchronous learning, peer instruction, immediate performance, conceptual transfer

## Abstract

**Introduction:**

Multiple-choice questions are widely used in higher education and online training because of their scalability. This study examines whether incorporating peer assessment, such as evaluating written explanations accompanying students’ answers, can increase students’ cognitive engagement and improve learning outcomes in asynchronous settings.

**Methods:**

One hundred undergraduate science students were randomly assigned to three groups and completed a series of conceptual questions on an online platform. Group A responded to standard multiple-choice questions. Group B additionally evaluated fictional peer explanations using agreement ratings. Group C additionally provided written feedback. All participants completed four experimental questions followed by five transfer questions. Completion time and performance at each phase were recorded.

**Results:**

Students in both peer assessment groups demonstrated significant gains between initial and revised answers. A linear regression revealed a significant effect of the peer assessment type on immediate performance, with written feedback benefiting low-performing students more markedly. However, no group outperformed the control in the transfer questions. Task duration increased significantly with peer assessment, particularly for the group providing written comments.

**Discussion:**

Embedding peer assessment appears to enhance immediate MCQ performance, especially when students are required to articulate written feedback; however, it did not improve transfer performance and significantly increased task duration. The absence of measurable gains in conceptual learning raises questions about the cost–benefit ratio of such interventions in time-constrained educational settings. Further research is warranted to delineate the conditions under which such interventions yield long-term benefits.

## 1 Introduction

Multiple-choice questions (MCQs) are widely employed in higher education due to their scalability, ease of administration, and suitability for both formative and summative assessment. Their efficiency makes them particularly well-suited for large cohorts and asynchronous learning environments such as online courses and revision platforms. Although multiple-choice tests are often criticized for promoting superficial engagement by emphasizing recognition over active retrieval, empirical studies have demonstrated that, when carefully designed, such assessments can support meaningful retrieval and enhance later recall (Clark et al., 2025).

Various instructional strategies have been developed to further enhance the cognitive impact of MCQs. Recently, requiring learners to justify their answers in multiple-choice tests in an online testing platform has been shown to enhance both immediate and delayed performance, likely through the activation of elaborative retrieval processes (Clark et al., 2025). Another prominent example is peer instruction, a method originally designed for synchronous classroom settings, which involves students answering a conceptual question individually, discussing their reasoning with peers, and then voting again (Crouch and Mazur, 2001; Mazur, 1997). This approach has been shown to improve learning outcomes by encouraging elaboration and co-construction of knowledge (Smith et al., 2011; Vickrey et al., 2015; Schell and Butler, 2018).

Building on these insights, asynchronous digital adaptations have emerged to extend the benefits of peer instruction beyond the classroom. Notably, tools such as myDALITE (Bhatnagar et al., 2016; Charles et al., 2019) and elastic (Andriamiseza et al., 2023; Parmentier and Silvestre, 2019) implement structured peer assessment workflows wherein learners respond to a conceptual MCQ providing a rationale to justify their choice, evaluate peer explanations, are given the opportunity to revise their initial answer, and receive expert feedback. These platforms aim not only to approximate the pedagogical value of live peer dialogue occurring during peer instruction within asynchronous formats, but also to address certain limitations inherent to synchronous face-to-face interactions. Notably, by anonymizing peer exchanges, these tools may reduce relational influence biases—where learners’ responses might otherwise be swayed by the identity or social status of their interlocutors rather than the quality of their arguments. Furthermore, by requiring learners to articulate written justifications, they support formative activities that mirror the demands of written justification often expected in summative assessments (Biggs, 1996).

However, according to the ICAP framework (Chi and Wylie, 2014), such activities may elicit constructive rather than interactive engagement, as learners generate explanations or evaluations without engaging in real-time dialogue or receiving peer counter argument — unlike the peer instruction method, in which students are required to actively engage in debate. While the ICAP framework provides a useful lens to classify the cognitive engagement elicited by different learning activities, it remains unclear whether asynchronous peer assessment—especially when lacking reciprocal interaction—elicits more deep processing than the usual MCQ format. Moreover, such interventions may impose an additional cognitive load on novice learners, potentially undermining their effectiveness despite increased engagement (Paas and Sweller, 2012).

The instructional design employed in these platforms constitutes a form of peer assessment tailored to the evaluation of explanations accompanying multiple-choice responses. A substantial body of research has demonstrated the pedagogical benefits of peer assessment, particularly when implemented in computer-mediated environments (Double et al., 2020; Li et al., 2020; Zheng et al., 2020). Peer assessment generally comprises two complementary components that both support learning: (1) evaluating others’ work and (2) receiving feedback from peers (Dmoshinskaia et al., 2021b). In the case of these platforms, however, only the evaluative component is enacted.

Despite promising theoretical foundations and encouraging preliminary findings, no rigorous empirical study has yet been conducted to assess the effectiveness of incorporating a peer assessment phase into multiple-choice testing. The present study seeks to address these gaps by examining the impact of integrating peer assessment into multiple-choice questions on immediate performance, conceptual transfer, and task duration, in comparison with the standard MCQ format.

## 2 Materials and methods

### 2.1 Participants and context

The study involved 100 undergraduate students enrolled in a third-year electricity course at a French engineering school. All participants were recruited from the same cohort and had previously completed instruction on the relevant conceptual topics. The activity was introduced to students as a voluntary online exercise designed both to help them prepare for an upcoming exam and to contribute to a pedagogical experiment. The instructor informed participants via email that the purpose of the experiment was to identify the most effective quiz design and that each participant would receive a slightly different version of the quiz. To encourage serious participation, students were instructed not to discuss the quiz content and students who completed the task received one bonus point on their final exam. Students had 1 week to complete the quiz, which had to be taken in a single uninterrupted session.

### 2.2 Experimental design

A between-subjects randomized controlled design was employed to compare three instructional conditions differing in the level of peer assessment involved. Each participant completed a sequence of nine multiple-choice questions (MCQs) on the online school’s Moodle platform. In all groups, the questions required conceptual reasoning rather than factual recall.

The students were randomly divided into three groups. The first four MCQs differed across groups as follow:

- Group A (control) completed standard MCQs with no peer-related components.
- Group B answered MCQs and then evaluated two fictional peer explanations, indicating their level of agreement on a Likert-type scale, and had a second chance to modify their answers.
- Group C performed the same task as group B but was also asked to write textual feedback for each evaluated explanation.

After completing all four questions, students proceeded to five transfer questions assessing conceptual learning. These were identical for all participants and did not include any peer evaluation or feedback components. The Moodle platform recorded completion time and response data.

The independent variable was the instructional condition, with three levels corresponding to the type of peer assessment activity: no peer interaction (group A), peer evaluation without feedback (group B), and peer evaluation with written feedback (group C). The primary dependent variables were:

1. The change in accuracy between initial and revised answers on the four conceptual MCQs (immediate performance),
2. Performance on the five transfer questions,
3. And the total task duration, as recorded by the Moodle platform.

We hypothesize that peer assessment will improve both immediate performance and performance on transfer items. Based on literature review, we also hypothesize that greater gains will be observed for students required to produce written feedback (Dmoshinskaia et al., 2021a). Hence, relative to both immediate performance and performance on transfer items:


C>B>A.

However, given that numerous studies have shown effects contingent on learners’ level of expertise (Kalyuga et al., 2003; Chen et al., 2017), a similar moderation effect may reasonably be expected in the present context.

### 2.3 Materials

The MCQs focused on core concepts in electricity and were designed to elicit common misconceptions in this domain (Knight, 2002; Wainwright, 2007). Each question included distractors representing typical erroneous reasoning. For peer evaluation in groups B and C, two fictional explanations were created for each question: one corresponding to the correct answer and one to a common incorrect choice. These were crafted by the instructional team based on literature on students misconceptions. They were written in a student-like style and typically consisted of one or two sentences. The use of fictional peer responses ensured standardization across participants and allowed for precise control over the nature of the feedback content. This design choice prevented variability associated with live peer explanations, while still simulating the cognitive processes involved in peer assessment. After each question, all students received identical corrective feedback written in a teacher-like style, typically comprising four to five explanatory sentences. Table 1 presents an example of a question asked, along with the students’ fictitious answers and the teacher’s explanation.

**Table 1:** Example of a conceptual multiple-choice question used in the experimental phase, including two fictional student explanations (one correct, one incorrect) and the standardized teacher feedback provided to all participants.

Component	Content
MCQ statement	In the circuit shown below, if the switch is turned off, the brightness of bulb A:brightness of bulb A: 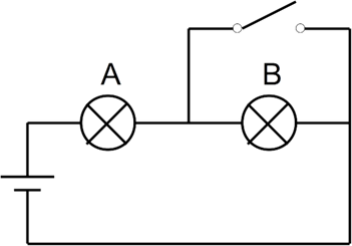 1. increases 2. remains constant 3. decreases
Correct fictional student’s explanation	[1] Since bulb B is short-circuited, all of the battery voltage is applied to the bulb terminal. The power dissipated is therefore greater, and the bulb shines brighter.
Incorrect fictional student’s explanation	[2] The current flowing through bulb B now flows through the short circuit. This does not affect bulb A.
Feedback	Correct answer: [1] In the initial circuit, with the two bulbs in series, the total resistance is 2R (R was the resistance of a single bulb). When the switch is closed, the equivalent resistance of the wire in parallel with bulb B becomes zero (bulb short-circuited). The total resistance is therefore R (a single bulb). The current supplied by the battery therefore becomes twice as high. Bulb A therefore shines brighter.

Text translated from French.

### 2.4 Procedure

Participants were randomly assigned to three experimental groups of similar size (group A: *n* = 37; group B: *n* = 33; group C: *n* = 30).

**Figure 1:**
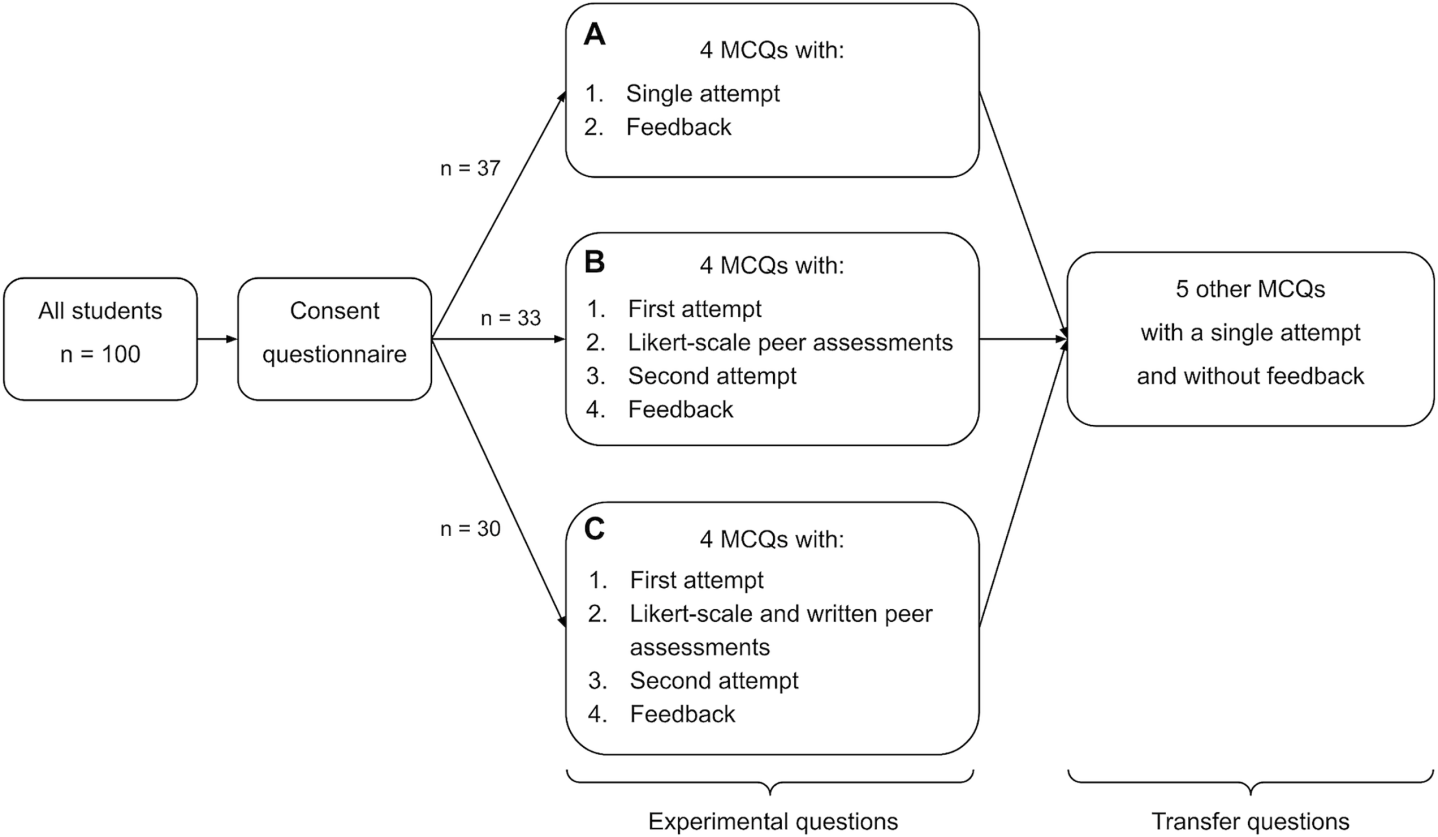
Schematic representation of the experimental procedure, showing the sequence of phases for each group (control, peer evaluation, and peer feedback),

After completing the consent questionnaire, each participant completed nine questions in total. The first four questions were experimental and presented in the format corresponding to the participant’s assigned group. The remaining five questions served to assess the potential transfer of learning. These were identical for all participants and did not include any peer evaluation or feedback components. The complete experimental procedure is shown in Figure 1. All activities were implemented using the Moodle platform. The system recorded completion time and response data.

In the control condition (group A), participants responded to each of the four conceptual multiple-choice questions (MCQs) following a standard two-phase structure: after reading a particular MCQ and viewing the associated circuit diagram, students selected one of the proposed answers and submitted their response (Phase 1). They then received corrective and explanatory feedback for this MCQ (Phase 2). They then answered another MCQ. After answering a particular MCQ, students could not go back to review the questions they had already answered.

In the peer evaluation condition (group B), each conceptual MCQ followed a four-phase structure. In Phase 1, participants submitted an initial answer to the question, identical to that in the control condition. During phase 2, they were asked to read two fictional student explanations: one aligned with the correct answer and one reflecting a common misconception. Students were asked to indicate their level of agreement with each explanation using a 4-point Likert scale (from “Totally agree” to “Totally disagree”). The students did not know which of the two answers was correct. In Phase 3, participants could revise or maintain their initial answer. And finally, in Phase 4 they received the corrective and explanatory feedback (identical to that in the control condition). This structure was repeated for each of the four experimental questions.

In the peer evaluation with feedback condition (group C), the structure was identical to group B during Phases 1, 2 and 4. However, in addition to rating their agreement with each peer explanation, students were asked to write a brief comment justifying their agreement or disagreement (“*What would you say to this student? Write a constructive comment explaining why you agree or disagree*”). A text box was provided for each explanation, prompting students to formulate a constructive response (*“I [agree/disagree] with you because…”*). This written feedback component was intended to elicit a higher level of cognitive engagement. After submitting both the ratings and comments, students proceeded to Phase 3 (revising their answer) and Phase 4 (feedback).

Note that for students in Groups B and C, corrective and explanatory feedback was provided only after the second attempt, ensuring that their answer revisions and evaluations of the fictional peer explanations were not influenced by the expert feedback.

After completing the four questions, all participants moved directly to the five transfer questions. These were structurally similar MCQs designed to assess conceptual transfer. No feedback was provided during the questions, and students could not revisit previous questions.

## 3 Results

### 3.1 Performance on experimental questions (immediate performance)

The average score on the first attempt across the initial four experimental questions was not significantly different across groups (group A: 42%, group B: 45%, group C: 48%; Kruskal-Wallis test: χ^2^ = 0.59; *df* = 2; *p =* 0.74), indicating no significant difference in baseline performance.

Participants showed a statistically significant improvement between the first and second vote both in group B (first vote: 45%; second vote: 53%; Wilcoxon signed-rank test: *W* = 12; 20 pairs of values were tied; *p =* 0.013; *d* = 0.48 [0.12; 0.84]) and group C (first vote: 48%; second vote: 62%; *W* = 0.00; 16 pairs of values were tied; *p* < 0.001; *d* = 0.84 [0.41; 1.25]). When comparing the second-vote performance, group C achieved a higher mean accuracy than group B (62% vs. 53%), but this difference was not statistically significant (Mann–Whitney: *U* = 423; *p =* 0.30; *d* = 0.35 [−0.24; 0.93]).

A linear regression was conducted to examine whether the relationship between first-attempt performance and second-attempt performance differed as a function of peer assessment condition (group B: evaluation only; group C: evaluation with written feedback). The analysis included the average score on the first attempt as a covariate, group as a fixed factor, and their interaction term. The Durbin–Watson test indicated no autocorrelation of residuals (autocorrelation = −0.052; *DW* = 2.10; *p =* 0.78), supporting the assumption of independence. The Q–Q plot showed approximate normality of residuals. The residuals-versus-fitted plot displayed no evident pattern or heteroscedasticity, supporting both linearity and homoscedasticity assumptions. Overall, key regression assumptions were met. The overall model was statistically significant (*F*(3, 59) = 47.8; *p* < 0.001), and accounted for approximately 71% of the variance in second-attempt scores (*R*^2^ = 0.71; adjusted *R*^2^ = 0.69).

The regression model revealed that first-attempt performance was a strong positive predictor of second-attempt performance (*β* = 0.91; *p* < 0.001), indicating that higher initial scores were associated with higher scores on the second attempt. The effect of the peer assessment condition was also significant: participants in group C (evaluation with written feedback) performed significantly better on their second attempt than those in group B (evaluation only), controlling for initial performance (*β* = 0.70; *p =* 0.025). The interaction between peer assessment condition and first-attempt performance approached significance (*p =* 0.089), indicating a possible trend toward differential effects of prior performance across groups. The negative direction of the coefficient (*β* = −0.24) suggests that the benefit of written feedback may be more pronounced for participants with lower initial scores.

**Figure 2:**
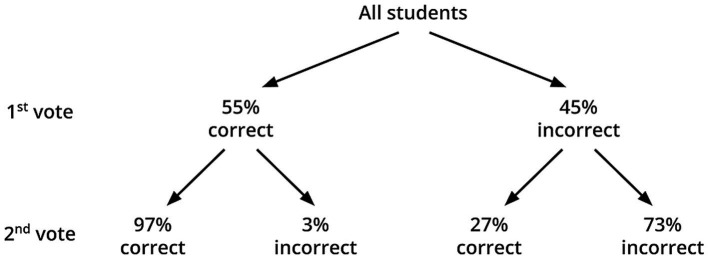
Response transitions between the first and second vote among all students in the peer assessment conditions (Groups B and C combined).

Figure 2 presents changes in student responses following the peer assessment phase. Data from groups B and C were combined, and means were calculated across the four experimental questions. On average, 55% of responses were correct on the initial vote. Among students who answered incorrectly in Phase 1, 27% revised their answer to the correct one in the second vote. Among students who answered correctly on the first attempt, only 3% selected an incorrect answer on the second attempt after evaluating the fictional peer explanations.

### 3.2 Performance on transfer task

Mean scores on the five transfer questions did not differ significantly between groups (group A: 49%, group B: 53%, group C: 52%; Kruskal-Wallis test: χ^2^ = 0.25; *df* = 2; *p =* 0.88), suggesting no measurable advantage of peer assessment strategies on this outcome.

A linear regression was conducted to predict performance on transfer MCQs from average first-attempt performance during training, peer assessment group (A, B, C), and their interaction. The model explained approximately 19% of the variance in exam scores (*R*^2^ = 0.19; adjusted *R*^2^ = 0.15). The main effect of the first-attempt performance approached significance (*p =* 0.054), suggesting a possible positive association between first-attempt performance and exam outcomes. However, group membership (group B or C versus group A) was not a significant predictor (*p =* 0.41 and *p* = 0.91 respectively), nor were the interaction terms between first-attempt performance and group (*p =* 0.21 and *p* = 0.82 respectively). These results indicate that, although better first-attempt performance may be weakly associated with higher exam scores, there is no evidence that this relationship differs meaningfully by peer assessment condition, despite the additional effort required in groups B and C.

### 3.3 Completion time and students participation

The distribution of response times follows roughly a log-normal distribution. The median time taken to complete the entire activity (consent form, experimental MCQs, and transfer MCQs) differed significantly between groups: 14 min 36 s for group A, 16 min 18 s for group B, and 24 min 48 s for group C (Kruskal-Wallis test: χ^2^ = 24; *df* = 2; *p* < 0.001). Quartiles are shown in Table 2. It is interesting to note that the 75th percentile of group A is smaller than the 25th percentile of group C. This lack of overlap in the interquartile ranges between groups A and C highlights a substantial shift in response behavior, indicating that participants in group C generally engaged with the task for a markedly longer duration than those in group A.

**Table 2:** Descriptive statistics (25th, 50th, and 75th percentiles) for task completion time (in minutes) across the three experimental groups.

Group	A	B	C
25th percentile	10.8	14.0	17.8
50th percentile	14.6	16.3	24.8
75th percentile	16.8	19.3	37.1

For group B and C, Likert scale assessments were completed in 95% of cases. The few missing assessments are mainly attributable to three students. Assuming that the increase in completion time for group B compared to group A is mainly due to the Likert scale assessment of contributions, a student can be estimated to take an average of 18 s to assess a contribution.

For group C, 80% of the text feedback forms were completed. The few missing assessments are mainly attributable to five students. The average length of these comments was 153 characters, with a standard deviation of 87. An example of average length is: “*I disagree. That will not change anything for light bulb A. See Ohm’s law: at the output of the short circuit, the intensity is the same as it was at the input*” (translated from French). Sixty-eight percent of the comments adhered to the suggested template (“I [agree/disagree] with you because…”) or a close variation thereof (e.g., “I totally agree with you because…”). Although the majority of students adhered to the template, a notable proportion deviated from the suggested format, which may reflect varying levels of engagement or understanding of the instructions. Assuming that the increase in completion time for group C compared to group B is mainly due to the writing of textual feedback, a student can be estimated to take an average of 1 min 20 s to write a feedback.

## 4 Discussion

This study investigated the impact of integrating peer assessment mechanisms into multiple-choice question tasks on immediate conceptual performance, transfer of learning, and task duration. By comparing traditional MCQs (group A) with two peer assessment conditions—evaluation only (group B) and evaluation with written feedback (group C)—we aimed to examine whether these interventions could enhance learning outcomes in an asynchronous digital context.

### 4.1 Impact on immediate performance

Consistent with prior research on peer instruction (Crouch and Mazur, 2001; Smith et al., 2009, 2011), our findings indicate that peer-related activities improve immediate performance on conceptual MCQs. Specifically, both peer assessment groups showed statistically significant improvements between initial and revised responses. Moreover, group C—whose participants were required to produce written feedback—exhibited higher gains, suggesting that this added cognitive effort may facilitate deeper engagement. Although the difference in second-attempt accuracy between group B and C did not reach statistical significance, the linear regression revealed a significant main effect of the group after controlling for baseline performance.

Importantly, the observed interaction between initial performance and instructional condition—though only marginally significant—points to a potential moderating role of prior knowledge. Students with lower initial scores appeared to benefit more from the feedback-writing task, a pattern that demonstrates a reversal of expertise effect (Kalyuga, 2007). This supports the hypothesis that lower-performing students may gain more from structured elaboration activities, which help scaffold their reasoning processes. This result aligns with findings by Dmoshinskaia et al. (2021a,b) who demonstrated that providing written comments—rather than evaluative ratings—leads to greater learning gains for peer reviewers, particularly among students with lower prior knowledge (Dmoshinskaia et al., 2021a).

Following peer assessment, 27% of initially incorrect responses were corrected, while only 3% of initially correct answers were changed to incorrect ones. This suggests that peer assessment had minimal negative impact on immediate performance: students who initially responded correctly were highly likely to maintain their correct answer. However, it is worth noting that the overall magnitude of change was smaller than typically observed in peer instruction studies in university settings. For initially incorrect responses, studies reported 59% of change in physics (Crouch and Mazur, 2001), 42% in biology (Smith et al., 2009) and 59 and 48% in computing (Porter et al., 2011). This rate was also higher for change from correct to incorrect: 13% in physics, 8% in biology, and 14 and 28% in computing (Porter et al., 2011; Crouch and Mazur, 2001; Smith et al., 2009). This discrepancy may be attributable to the asynchronous nature of the task, which lacks the dialogic interactivity characteristic of live peer discussions. According to the ICAP framework (Chi and Wylie, 2014), peer instruction is interactive, whereas peer assessment may only elicit constructive engagement without true interaction. Although reviewers may construct additional knowledge beyond the content of the (fictional) peer explanation, the absence of turn-taking prevents interactive dialogue. Thus, peer assessment can be conceptualized as a hybrid form of engagement that falls between constructive and interactive modes as described in the ICAP framework. These findings support the growing body of research suggesting that giving feedback, rather than merely receiving or rating it, yields stronger cognitive benefits for the feedback provider (Dmoshinskaia et al., 2021a, 2021b).

In sum, the peer assessment phases—particularly when requiring written feedback—appeared to foster meaningful cognitive engagement and improved immediate performance. However, whether these short-term gains translate into deeper conceptual learning remains an open question. We now turn to the analysis of performance on transfer questions to assess whether such benefits were sustained beyond the immediate task.

### 4.2 Absence of transfer gains

Contrary to expectations, neither peer assessment condition led to significant improvements of performance in the transfer questions. Despite better immediate performance, no group outperformed the control condition on the evaluation questions. This finding diverges from literature on peer instruction, which frequently reports transfer gains (Smith et al., 2009, 2011). Although the absence of reciprocal, dialogic interaction may partly explain the limited effects on transfer, we note that the peer assessment tasks implemented here still qualify as constructive engagement under the ICAP framework (Chi and Wylie, 2014). Learners were required to evaluate and comment on peer explanations, thus actively generating new knowledge beyond mere reception. According to ICAP, such constructive activities are expected to foster deep learning and transfer, even in the absence of interactive dialogue. Thus, the absence of transfer effects in this study likely reflects the influence of additional limiting factors beyond the level of cognitive engagement alone.

One possibility is that the intervention’s short duration (15–25 min) was insufficient to support meaningful conceptual restructuring.

Second, although peer assessment was associated with improvements in immediate performance, its lack of impact on transfer might stem from the limited effectiveness of the peer evaluation process itself. Only 27% of students who initially answered incorrectly were able to revise their answer correctly after evaluating peer explanations. This suggests that, in the majority of cases, students may have failed to recognize the correct reasoning, especially in the absence of peer confrontation or justification, potentially reinforcing their initial misconceptions rather than correcting them—an effect documented in prior research (Rittle-Johnson and Loehr, 2017). As a result, the potential benefits of the activity for students who answered correctly at first and were able to identify correct explanations may have been counterbalanced by negative effects on those holding incorrect conceptions, thereby neutralizing overall gains in transfer.

Another plausible explanation for the absence of transfer gains lies in the limited scaffolding provided for generating high-quality peer feedback and engaging in deeper reasoning. While students in group C were prompted to justify their evaluations, they received no specific guidance on what constitutes effective feedback or how to identify key conceptual features in peer explanations. This lack of structure may have hindered their ability to engage in elaborative processing, especially for students with lower prior knowledge.

Finally, the fictional nature of peer contributions may have limited authenticity and thus limited engagement and motivation in the evaluation effort. According to the argumentative theory of reasoning (Mercier and Sperber, 2011; Mercier and Landemore, 2012), reasoning evolved not primarily to discover truth in isolation, but to argue effectively in a social context where one’s views are challenged and defended. Evaluating fictional, anonymized student arguments in a written format does not elicit the same epistemic vigilance or motivational engagement as a live, oral debate with real peers. Consequently, without the pressure to convince or to respond to challenges, learners may not engage in the kind of strategic, effortful reasoning necessary to revise or consolidate their conceptual understanding at a level that supports transfer, as observed during Peer Instruction (Smith et al., 2009, 2011).

### 4.3 Completion time and implications for practice

The addition of peer assessment, particularly written feedback, significantly increased task duration. While some of this increase may reflect greater cognitive engagement, no transfer benefits were observed. This raises questions regarding the instructional efficiency of elaborative peer assessment tasks, defined here as the ratio between learning gains and invested learner time (van Gog and Paas, 2008). In the absence of significant transfer effects, the cognitive effort and temporal cost required by Group C may not be justifiable in typical time-constrained learning environments. However, such tasks may be justified in two specific contexts. First, when fostering students’ evaluative judgment is an explicit instructional goal—for example, in developing critical thinking or peer review skills—this form of engagement holds intrinsic value. Second, if teachers can access students’ peer evaluations and written justifications, these responses may serve as formative indicators of conceptual understanding. In this case, the activity supports instructional adaptation, a key factor in student learning (Chen et al., 2020; Sadler et al., 2013). Thus, the cost–benefit ratio of peer assessment depends not only on its alignment with instructional goals, but also on the extent to which teachers can exploit students’ evaluative outputs to inform and adapt their teaching.

### 4.4 Limitations and future directions

While previous results raise important considerations regarding the pedagogical efficiency of asynchronous peer assessment, they must be interpreted with caution. We outline in this section several methodological and contextual limitations that may have influenced the observed outcomes and suggest directions for future research.

The first limitation concerns statistical power. Although the overall sample size (*N* = 100) was adequate for detecting large effects, it may have been insufficient to reliably detect small to moderate effects, particularly on transfer performance. A post-hoc power analysis using G*Power (one-way ANOVA, three groups, *α* = 0.05) indicates that the current design achieves 95% power to detect a large effect size (*f* = 0.40), and 80% power for an effect size of *f* = 0.32. Given that *f* = 0.25 is typically considered a medium effect (Cohen, 1988), the study may have been underpowered to detect more subtle differences between conditions. This limitation likely contributes to the absence of significant effects observed on transfer tasks.

Moreover, the study’s generalizability is limited by the sample, which consisted solely of students from a single French engineering course. Cultural, disciplinary, and institutional factors may influence how students engage with peer assessment, and the observed effects may not hold in other contexts. For instance, the effectiveness of structured peer feedback may be shaped by students’ disciplinary epistemologies, their familiarity with peer learning formats, and culturally mediated expectations regarding feedback and collaboration. Future research should replicate this study across diverse academic disciplines and educational settings to assess the robustness and external validity of the findings.

Additionally, the transfer test was administered immediately after the experimental phase. On the one hand, it is possible that delayed effects may have occurred but were not captured; on the other hand, only long-term effects are of practical relevance for instructional design and learning outcomes. Future studies should therefore incorporate delayed post-tests to better assess retention and potential delayed learning gains.

The use of fictional peer explanations, while allowing for experimental control, may have reduced students’ cognitive engagement with the task. Incorporating real peer-generated content could increase ecological validity and promote deeper processing by enhancing the perceived relevance and authenticity of the activity. Future studies should examine whether using authentic student responses enhances the effectiveness of peer evaluation tasks.

Instructional designers should carefully design peer tasks to mitigate the risk of reinforcing misconceptions through exposure to incorrect reasoning. Future studies should investigate expertise-related effects, including adaptive peer assessment models that adjust task complexity or guidance based on learners’ initial expertise, and could also explore whether introducing minimal forms of asynchronous dialogue—such as structured peer reply chains—might bridge the gap between constructive and interactive engagement while preserving flexibility.

Another promising direction involves introducing structured peer dialogue or minimal asynchronous interaction, such as reply chains, to approximate interactive engagement. While the current design elicited primarily constructive engagement through individual feedback, incorporating opportunities for learners to respond to peers’ comments could foster deeper cognitive processing by promoting argumentation, clarification, and perspective-taking. In asynchronous conditions, a conversational AI agent could be used to simulate a fictitious peer who authored the explanation to be evaluated. Future studies should investigate whether such minimal forms of interaction enhance the learning benefits of asynchronous peer assessment.

## Data Availability

The datasets presented in this study can be found in online repositories. The names of the repository/repositories and accession number(s) can be found at: doi: 10.17605/OSF.IO/X8MVU.
